# Clinical Ascites and Emergency Procedure as Determinants of Surgical Risk in Patients with Advanced Chronic Liver Disease

**DOI:** 10.3390/jcm14041077

**Published:** 2025-02-08

**Authors:** Lidia Canillas, Amalia Pelegrina, Fawaz Wasef León, Aina Salis, Elena Colominas-González, Antonia Caro, Juan Sánchez, Juan Álvarez, Fernando Burdio, Jose A. Carrión

**Affiliations:** 1 Department of Medicine and Life Sciences, Universitat Pompeu Fabra, 08003 Barcelona, Spain; lcanillas@psmar.cat (L.C.); apelegrina@psmar.cat (A.P.); fawazwasef.leon01@estudiant.upf.edu (F.W.L.); aina.salis01@estudiant.upf.edu (A.S.); ecolominasgonzalez@psmar.cat (E.C.-G.); jsanchezp@psmar.cat (J.S.); jcalvarez@psmar.cat (J.Á.); fburdio@psmar.cat (F.B.); 2 Liver Section, Gastroenterology Department, Hospital del Mar, 08003 Barcelona, Spain; acaro@psmar.cat; 3 IMIM (Hospital del Mar Medical Research Institute), 08003 Barcelona, Spain; 4 Department of General Surgery, Hospital del Mar, 08003 Barcelona, Spain; 5 Department of Medicine, Universitat Autònoma de Barcelona, 08003 Barcelona, Spain; 6 Pharmacy Department, Hospital del Mar, 08003 Barcelona, Spain; 7 Abdominal Unit, Radiology Department, Hospital del Mar, 08003 Barcelona, Spain; 8 Anesthesia Department, Hospital del Mar, 08003 Barcelona, Spain

**Keywords:** advanced chronic liver disease, cirrhosis, portal hypertension, ascites, postoperative risk, surgery, mortality, emergent

## Abstract

**Background:** Liver function and the presence of portal hypertension, as well as the urgency and type of surgery, are prognostic factors in advanced chronic liver disease (ACLD) patients undergoing extrahepatic major surgeries. Emergent surgery in ACLD patients has 4–10 times higher mortality rates than elective surgery. However, perioperative management improvements have been made in recent years. **Methods:** This is a retrospective, observational, and unicentric study of 482 patients with ACLD who underwent major surgery from 2010 to 2019. We compared baseline characteristics and postoperative mortality according to the presence of ascites, the emergency, and the surgery period. **Results:** In total, 140 (29%) patients had ascites, and 191 (39.6%) underwent urgent surgeries. The 90-day mortality was 2.8-fold higher in patients with ascites [HR (95%CI) 2.8 (1.6–5.0); *p* = 0.001] and 3-fold higher in urgent surgeries [3.0 (1.6 − 5.5); *p* < 0.001)]. Urgent surgeries in patients with ascites revealed the highest mortality risk [6.3 (2.7–14.8); *p* < 0.001)], which persisted in current (2015–2019) surgeries [12.8 (2.9–56.5); *p* = 0.001)]. Portal hypertension was meaningful in patients undergoing abdominal surgery. **Conclusions:** ascites and emergent surgery increase the mortality risk of patients with ACLD despite the recent perioperative improvements.

## 1. Introduction

In recent years, there has been an increase in surgery indications in patients with advanced chronic liver disease (ACLD) or cirrhosis due to the rise in its prevalence and life expectancy. Hepatic decompensation itself is already a poor prognostic factor that impacts the natural history of ACLD, regardless of the need for surgical intervention [1]. Patients with chronic disorders are at risk of suffering from acute pathologies or oncological diseases that require surgical interventions in the absence of an effective, less invasive alternative. Major surgery in ACLD patients is associated with an increased risk of mortality compared to the general population [2]. Surgery is an event that could impact on liver function, leading to liver-related events, acute kidney injury (AKI), and infections [3,4]. The increased surgical morbimortality risk in patients with ACLD is multifactorial and associated with (1) the presence of clinically significant portal hypertension (CSPH), (2) the liver function grade, (3) the comorbidities, (4) the emergency nature of the surgery, and (5) the type of surgery [3,4,5]. The Child–Turcotte–Pugh (CTP) score and the Model for End-Stage Liver Disease (MELD) are widely used to assess liver function. Child–Pugh B and C patients showed higher morbimortality risk after abdominal [6,7] and other surgeries [8]. The higher the MELD score, the higher the surgical risk [9,10]. Urgent surgery has shown a 4–10 times higher mortality compared to elective surgery [11]. Open-major abdominal and cardiovascular surgeries carry the highest mortality risk [12].

Significant improvements have been made in the perioperative management of patients with ACLD in recent decades. Direct interventions on alcohol-, hepatitis C virus-, and hepatitis B virus-related ACLD have demonstrated efficacy in improving liver function and patient outcomes [13]. In recent years, treatment with non-cardioselective beta-blocker drugs (carvedilol or propranolol) has become widespread in patients with signs of CSPH, as it has demonstrated its benefit in reducing the risk of decompensation and mortality [14,15]. Moreover, new scores evaluating mortality risk have been recently validated in patients with ACLD. The VOCAL-Penn [16] and NSQIP [4,17] scores have shown good accuracy in predicting mortality and clinical decompensation after major surgeries.

Hence, this study aimed to compare mortality between patients (1) with or without ascites, (2) who have had elective or emergent surgery, and (3) undergoing recent or past surgeries.

## 2. Materials and Methods

### 2.1. Study Design

A single-center retrospective study was performed at Hospital del Mar (Barcelona). A surgeon (A.P.) and two hepatologists (L.C., J.A.C.) evaluated patients with any diagnosis of chronic liver disease who underwent surgery between January 2010 and December 2019, detected through the hospital registry. All patients with ACLD who underwent major surgery were included. A previous publication detailed the identification of the study population [4].

A careful evaluation of the electronic data of the radiological diagnostic criteria (J.S.) was considered for the study. Patients with a risk factor for liver disease were diagnosed with ACLD if they presented any of the following: (1) liver stiffness measurement (LSM) by transient elastography > 15 kPa, (2) ultrasound or computerized tomography signs of ACLD, (3) platelets <150,000/uL and splenomegaly (≥13 cm), and/or (4) gastroesophageal varices (GOV) in upper gastrointestinal endoscopy.

The ethical committee of our institution, ‘Comitè Ètic d’Investigació Clínica-Parc de Salut Mar,’ approved the protocol (study reference 2020/9640) according to the ethical guidelines of the 1975 Declaration of Helsinki.

### 2.2. Study Variables and Definitions

We collected demographic data, and observed cardiovascular comorbidities, ASA scale values, the etiology of ACLD, liver and renal function, a noninvasive evaluation of CSPH, and the presence of ascites 30 days before surgery. The liver stiffness measurement (LSM; kPa) using FibroScan^®^ (Echosens, Paris, France) was included for patients without ascites 30 days before surgery [18]. After surgery, we observed liver and renal function, ascites decompensation, the presence of primary and secondary bacterial peritonitis, hospital stay, and mortality at 30 and 90 days from surgery.

We compared the patients according to their distribution in two equivalent periods: past (between 2010 and 2014) and present (between 2015 and 2019).

Ascites was graded as grade 1 (mild, only detected on ultrasonography), grade 2 (moderate, clinically managed with diuretics), and grade 3 (severe, which needs paracentesis) [19]. Grades 2 and 3 were considered clinical ascites.

We considered emergency surgery as those indicated by any urgent medical condition requiring attention in less than 24 h [20]. We included the same surgery location as the VOCAL-Penn [16]. To simplify the analysis, we grouped surgeries into abdominal (open and laparoscopic) and non-abdominal (abdominal wall, vascular, major orthopedic surgery, and cardiothoracic).

Postoperative comorbidity was evaluated by collecting the liver-related events (LREs) at 90 days, such as changes in ascites (new onset ascites, and worsening of previous grade), hepatic encephalopathy, gastrointestinal hemorrhage due to portal hypertension, and/or peritonitis infection. Worsening of ascites was regarded as cases of a change from grade 1 to grade 2 and from grade 2 to grade 3. Improvement of ascites was defined as the absence of ascites in patients with any grade 2 or 3 ascites before surgery. We defined AKI as described by the Kidney Disease Improving Global Guidelines [21].

### 2.3. Statistical Analysis

Continuous data were expressed as medians and interquartile range [IQR: percentile 25th–75th] and categorical variables as frequencies and percentages. Baseline characteristics of patients were compared with chi-square and U-Mann–Whitney as appropriate according to the presence or absence of ascites, the urgency of surgery, and the period of study. Variables related to 30-day mortality in patients undergoing abdominal and non-abdominal surgeries were assessed by univariate analysis with chi-square and U-Mann–Whitney tests. We performed survival Kaplan–Meier curves and Cox proportional hazard models to compare 90-day mortality between groups. The VOCAL-Penn’s discriminative capacity was assessed using c-statistic (area under the curve) and its 95% confidence interval (95%CI). The c-statistics were compared between groups with DeLong’s and Clarke-Pearson’s non-parametric method.

All the analyses were 2-tailed. Statistical analysis and graphs were performed using STATA V18.0 (StataCorp LLC., College Station, TX, USA).

## 3. Results

### 3.1. Baseline Characteristics of Patients

After a careful evaluation of the described radiological diagnostic criteria, 30 patients were excluded for the following reasons: absence of radiological criteria to establish the diagnosis of ACLD (21/512; 4.1%), congestive liver disease (4/512; 0.8%), metastatic liver (2/512; 0.4%), and exudative ascites (3/512; 0.6%).

Characteristics of included patients with ACLD (*n* = 482) are detailed in Table 1. Among the patients, 140 (29.0%) had clinical ascites: 68 (48.6%) grade 2 and 72 (51.4%) grade 3. Patients with ascites were more frequently ASA-IV (28.6% vs. 9.7%, *p* < 0.001). Liver function according to the levels of bilirubin, albumin, INR, MELD-Na, and CTP class was significantly better in patients without ascites. The median LSM in patients without ascites was 15.7 kPa, and 93 (47.2%) of 197 with available LSM showed an LSM < 15 kPa with at least one of the inclusion criteria: thrombocytopenia and splenomegaly (n = 28, 30.1%), ultrasonographic signs of ACLD (n = 82, 88.2%), and GOV (n = 33, 35.5%). Patients with ascites were more frequently operated on with abdominal wall surgery (39.3% vs. 15.8%, *p* < 0.001) but less with laparoscopic abdominal surgery (7.9% vs. 21.4%, *p* < 0.001). Among patients with ascites undergoing abdominal wall surgery (n = 55), the procedure was performed urgently in 67.3% (n = 37).

We observed that 191 (39.6%) surgeries were performed urgently (Table 2), and the most frequent locations were abdominal (43%), major orthopedic (25%), and abdominal wall (22%). Patients who underwent urgent surgery showed similar rates of GOV compared to elective surgery (64.6% vs. 58.4%; *p* = 0.214). However, patients in emergent situations were more frequently decompensated with ascites (39.3% vs. 18.6%; *p* < 0.001) and had worse liver function according to CTP≥ B7 (46.5% vs. 19.5%, *p* < 0.001) and MELD-Na (14 vs. 10, *p* < 0.001).

Patients undergoing current surgeries (2015–2019) were younger (median age 64 vs. 68 years, *p* = 0.004), and open abdominal surgeries were less frequently performed (17.3% vs. 30.6%, *p* = 0.001). Liver function, bilirubin, albumin, INR, and MELD-Na values were better in current surgeries, and we did not find differences between ASA categories. In the latter period, the median platelet value was higher (140 vs. 119 × 10^3^/uL; *p* < 0.001). However, no differences were found regarding the prevalence of Child–Pugh≥ B7 (26.1% vs. 34.2%, *p* = 0.075), ascites 30 days before surgery (26.5% vs. 32.0%; *p* = 0.189), and GOV (62.9% vs. 58.4%; *p* = 0.357) (Supplementary Table S1).

The oncological indication was more prevalent in patients without ascites (non-ascites: 15.2% vs. ascites: 7.1%; *p* = 0.016) and in elective surgeries (elective: 19.2% vs. urgent: 3.1%; *p* < 0.001); no differences were found between both periods (2010–2014: 14.4% vs. 2015–2019: 11.5%; *p* = 0.337).

### 3.2. Hospital Stay and Liver-Related Events After Surgery

Hospital stay (days) was higher [median (p25–p75)] in patients with ascites [13 (6–23)] than in those without ascites [7 (4–16)] (*p* < 0.001), and in emergent surgery [11 (6–20)] than in elective [7 (3–16)] (*p* < 0.001). However, the hospital stay decreased from 10 (5–19) days in past surgeries (2010–2014) to 7 (3–16) days in current surgeries (2015–2019) (*p* = 0.004).

LREs were observed in 160 (33.2%) patients. The impact of transjugular intrahepatic portosystemic shunt (TIPS) was unassessed because of the small number for its presence (n = 3). Among patients with ascites (n = 140) and emergent surgery (n = 191), 63.6% and 56.0% developed LREs at 90 days, respectively. Bacterial peritonitis was more frequent in patients with ascites (22.9% vs. 6.1%, *p* < 0.001) and in patients undergoing emergent surgeries (17.8% vs. 6.5%; *p* < 0.001).

Patients without ascites (n = 342) developed it after surgery in 19% (n = 65) of cases. In patients with ascites (n = 140), the surgery worsened its grade by 16.4% (n = 23). Patients with ascites developed AKI more frequently (47.4% vs. 27%) (*p* < 0.001) and more severely (grade 1B: 10.4%, grade 2: 14.1%, and grade 3: 12.6%) than those without ascites (grade 1B: 9%, grade 2: 5.1%, and grade 3: 5.5%) (*p* = 0.001) (Table 1). Patients with ascites improvement were more frequently treated with non-selective beta-blockers (37%) compared to those with no change/worsening (16.9%) (*p* = 0.008), and no difference in albumin levels was found (3.7 g/dL vs. 4 g/dL; *p* = 0.051). The three patients undergoing TIPS controlled the ascites with low doses of diuretics.

Patients who underwent emergent surgeries compared to elective had more frequent LREs (56.0% vs. 18.2%, *p* < 0.001), ascites (20.9% vs. 8.6%; *p* < 0.001), worsening of ascites (7.3% vs. 3.1%; *p* < 0.001), AKI (45.7% vs. 24.2%; *p* < 0.001), and bacterial peritonitis (17.8% vs. 6.5%; *p* < 0.001) (Table 2).

Patients on current surgeries compared to previous ones developed lower rates of LREs (26.9% vs. 40.5%; *p* = 0.002), ascites (10.4% vs. 17.1%; *p* = 0.031), and bacterial peritonitis (6.9% vs. 15.8%; *p* = 0.002). No differences between periods were found regarding the worsening of ascites (3.9% vs. 5.9%; *p* = 0.302) and the prevalence of AKI (32.9% vs. 33.5%; *p* = 0.896) (Supplementary Table S1).

### 3.3. Mortality Risk and Survival

Observed mortality (events, %) at 30 and 90 days was 6.4% (31/482) and 9.5% (46/482), respectively. Mortality at 30 days was 8.1% (16/197) in abdominal surgeries and 5.3% (15/285) in non-abdominal surgeries (*p* = 0.209) [4.6% (5/109) in the abdominal wall and 7.3% (9/123) in major orthopedic cases]. Mortality at 90 days was 11.7% (23/197) in abdominal and 8.1% (23/285) in non-abdominal surgeries (*p* = 0.185) [7.3% (8/109) in abdominal wall and 10.6% (13/123) in major orthopedic cases]. Postoperative mortality univariate analysis is depicted in Supplementary Table S2. The deceased patients after abdominal surgery were more frequently ASA-IV (75% vs. 18.2%; *p* < 0.001), had cardiovascular comorbidity (history of acute myocardial infarction, heart failure, or peripheral vasculopathy), underwent open surgery (93.8% vs. 54.1%; *p* = 0.002) and were submitted in an emergent condition (68.8% vs. 42%; *p* = 0.039). On univariate analysis, liver function and nutritional variables were related to mortality in both types of surgeries. However, variables associated with portal hypertension such as ascites and GOV were related to mortality only in abdominal surgeries (Supplementary Table S2).

**Figure 1:**
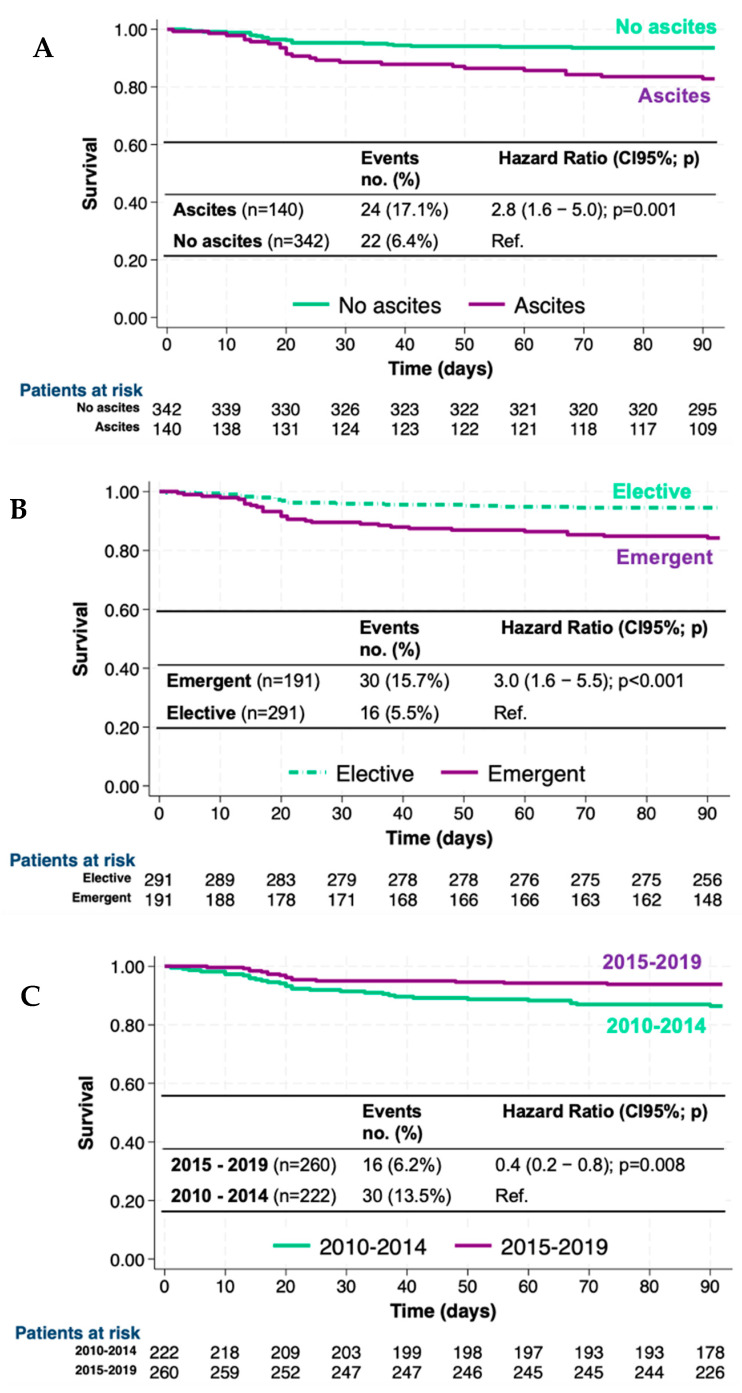
Survival curves (Kaplan–Meier) and 90-day mortality risk (HR) according to (**A**) ascites (presence vs. absence), (**B**) emergency of surgery (emergent vs. elective), and (**C**) period (2010–2014 vs. 2015–2019). HR: Hazard Ratio; 95%CI: 95% confidence interval.

Mortality at 90 days in patients with ascites was 17.1% (24/140) compared to 6.4% (22/342) in those without ascites (*p* < 0.001). Survival (1—mortality) curves according to ascites (absence vs. presence) are depicted in Figure 1A. The 90-day mortality risk (Hazard Ratio, HR) in patients with ascites was 2.8-fold higher than in patients without ascites [HR (95%CI) 2.8 (1.6–5.0); *p* = 0.001] (Figure 1A).

Mortality at 90 days in emergent surgeries was 15.7% (30/191) and in elective was 5.5% (16/291), respectively (*p* < 0.001). Survival curves according to the urgency of surgery are shown in Figure 1B. Therefore, 90-day mortality risk was 3 times higher in emergent surgeries compared to elective surgeries [3.0 (1.6–5.5); *p* < 0.001] (Figure 1B).

Observed mortality at 90 days in current surgeries (2015–2019) was significantly lower, 6.2% (16/260), than in past surgeries (2010–2014), 13.5% (30/222) (*p* = 0.006). Survival curves according to the period of surgery are depicted in Figure 1C. So, 90-day mortality risk decreased by 60% in current surgeries compared to previous surgeries [0.4 (0.2–0.8); *p* = 0.008] (Figure 1C).

### 3.4. Survival and Mortality Risk in Different Groups of Patients, Surgeries, and Periods

**Figure 2:**
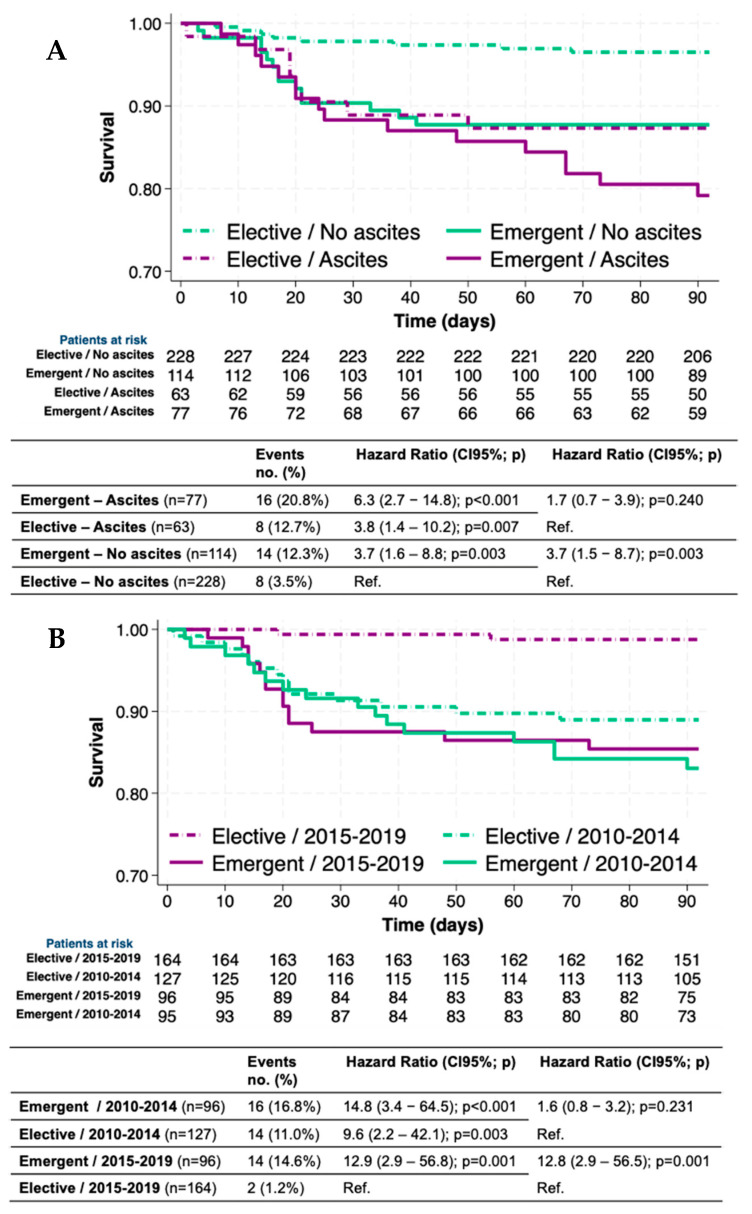
Survival curves (Kaplan–Meier) and 90-day mortality risk (HR) according to (**A**) emergency level of surgery (emergent vs. elective) and ascites (presence vs. absence), and (**B**) emergency level of surgery (emergent vs. elective) and period (2010–2014 vs. 2015–2019). HR: Hazard Ratio; 95%CI: 95% confidence interval.

Observed postoperative mortality ranged from 3.5% (8/228) in patients without ascites undergoing elective surgeries to 20.8% (16/77) in those with ascites operated on under emergent conditions. Patients with ascites operated on under emergent situations showed the highest 90-day mortality risk [6.3 (2.7–14.8); *p* < 0.001], followed by those with ascites operated on electively [3.8 (1.4–10.2); *p* = 0.007] and those without ascites operated on emergently [3.7 (1.6–8.8; *p* = 0.003]. Therefore, patients without ascites operated electively showed the lowest risk of mortality [Ref.] (Figure 2A).

When evaluating the interaction between the urgency of surgery and the period, postoperative mortality ranged from 1.2% (2/164) in current (2015–2019) elective surgeries to 16.8% (16/96) in past (2010–2014) emergent surgeries. Importantly, the 90-day mortality risk difference between emergent and elective surgeries was greater in current (2015–2019) [12.8 (2.9–56.5); *p* = 0.001] than in past (2010–2014) surgeries [1.6 (0.8–3.2); *p* = 0.231]. When comparing the periods in elective surgeries, we observed a higher 90-day mortality in the past (2010–2014) [9.6 (2.2–42.1); *p* = 0.003] than in current surgeries (Figure 2B).

### 3.5. VOCAL-Penn Diagnostic Accuracy in Patients with Portal Hypertension

**Figure 3:**
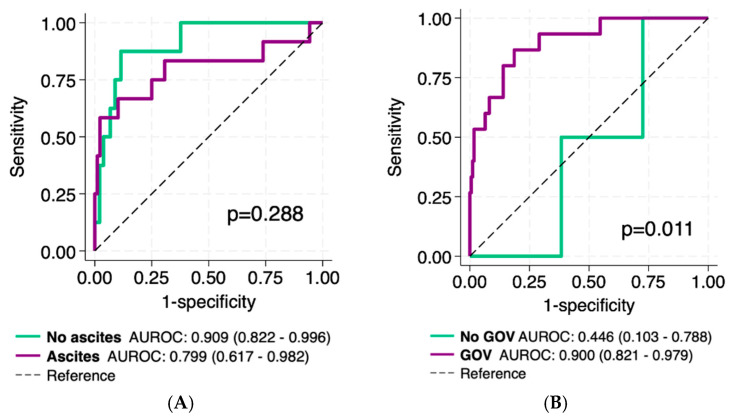
Comparison of the VOCAL-Penn diagnostic accuracy (ROC curves) to predict 30-day postoperative mortality according to (**A**) ascites (presence vs. absence), and (**B**) GOV (presence vs. absence).

The VOCAL-Penn discriminative capacity (c-statistic, 95%CI) for 30-day postoperative mortality was 0.799 (0.617–0.982) in patients with ascites and 0.909 (0.822–0.996) in those without ascites (*p* = 0.288) (Figure 3A). In contrast, the discriminative capacity of VOCAL-Penn was 0.446 (0.109–0.788) in patients without GOV compared to 0.900 (0.821–0.979) in those with GOV (*p* = 0.011) (Figure 3B).

## 4. Discussion

Previous studies have described a mortality rate 4–10 times higher in patients with cirrhosis undergoing urgent surgeries compared to elective surgeries [2]. Our study evaluating patients with and without ascites who underwent major surgeries has demonstrated a lower mortality risk but one still 3 times higher in emergent surgeries compared to elective. Patients undergoing elective surgeries in the current period (from 2015 to 2019) showed the lowest mortality. Several reasons can explain this observation. First, the selection of patients with better liver function and lower surgical risk after using current validated scores [4,16,17]. Second, the inclusion of minimally surgically invasive techniques, such as robotic and laparoscopic techniques, in current elective surgeries, but which are much more difficult in emergency situations. Third, the significant progress in the perioperative management of CSPH, hepatic decompensation, AKI, and infections in current clinical practice [22]. In contrast, some acute complications such as hemodynamic instability or sepsis can explain the high mortality in urgent surgeries.

Our study has shown that patients with ascites had worse liver function, and required oftentimes surgeries in emergent situations. Abdominal wall surgeries are frequent in decompensated ACLD patients because ascites has been described as a major risk factor for developing abdominal hernia [23]. Inguinal and umbilical hernias are often symptomatic, especially in patients with ascites and who have usually undergone urgent surgery due to hernia complications. A prospective observational study including patients with ascites (n = 30) has shown a 30-day postoperative mortality of 7% [24]. In consonance, we found a 30-day mortality of 4.6% in abdominal wall surgeries, for half of patients with ascites, and for two-thirds in an urgent setting. Only one randomized controlled trial has compared a conservative treatment to the elective repair of umbilical hernia in patients with ascites. Despite the low number of patients, it suggested that elective umbilical hernia repair can be safe in patients with ascites [25]. Based on these results, abdominal wall surgery should be considered even in patients with ascites, especially in experienced centers with a multidisciplinary approach to early management of portal hypertension and ascites [26].

Patients without ascites showed the greatest difference in mortality between urgent and elective surgeries compared to those with ascites before surgery. This finding may be due to different reasons: (1) the wide range of LSM in patients without ascites, from 11.2 to 23.6 kPa, indicating a high risk of CSPH, LREs, and mortality after an emergent surgery, (2) the low predictive capacity of VOCAL-Penn in patients without portal hypertension or GOV, (3) the different prevalence of abdominal surgery, one of the highest-risk surgeries, performed less frequently if patients had ascites; and (4) the lower incidence of intestinal resection in patients with ascites.

Finally, variables related to portal hypertension such as ascites and the presence of GOV were associated with 30-day mortality only in abdominal surgeries. Therefore, patients with ACLD undergoing abdominal surgeries could benefit from the intensification of the etiological treatment (alcohol withdrawal, antiviral drugs, etc.), the improvement of their nutritional status, and the evaluation of portal hypertension [27]. A non-invasive evaluation of CSPH could be beneficial to promptly diagnose and treat portal hypertension reserving hepatic venous pressure gradient (HPVG) measurement and TIPS use for most severe situations [5].

Our study has some limitations. Firstly, it was performed in a single center, but this large cohort of patients was well characterized by surgeons, anesthesiologists, radiologists, and hepatologists. Secondly, the retrospective design of the study does not allow us to know whether the worse liver function in patients undergoing urgent surgery was related to acute complications such as uncontrolled sepsis or patient selection; it is difficult to know whether patients with ACLD with non-oncological surgery were selected for elective surgery only if they had good liver function, while those with worse liver function or clinical decompensation only underwent urgent surgery in case of acute complications. Finally, the low number of deceased patients at 30 days did not allow us to perform a multivariate analysis. However, we have found that the predictive capacity of the VOCAL-Penn measure decreased in patients without GOV. Therefore, it is important to explore whether their diagnostic accuracy could be improved including other variables associated with portal hypertension.

In contrast, our study has important strengths and findings. It assessed the postoperative risk of patients with ACLD in three important situations (ascitic decompensation, emergency context, and period-based change) in a large and well-characterized cohort of patients. We found that (1) patients with ascites had 2.8-times-higher surgical mortality risk, (2) the risk of mortality for urgent surgeries is still 3 times higher than elective surgeries, (3) these differences persist in current emergent surgeries despite the progress in the perioperative management of patients with ACLD, (4) the evaluation and treatment of portal hypertension are meaningful in patients undergoing abdominal surgery, and (5) the predictive capacity of VOCAL-Penn decreased in patients without GOV.

## 5. Conclusions

The risk of mortality in patients with advanced chronic liver disease undergoing surgery has improved for elective but persisted for urgent situations over the years. The increasing demand for surgery forces us to explore new non-invasive methods to identify portal hypertension and surgical risk, establish novel strategies, and perform different algorithms for deciding the best time of intervention in compensated and decompensated patients. A multidisciplinary approach is imperative in decompensated patients, especially before emergent situations, to reduce mortality and complications.

## Figures and Tables

**Table 1:** Baseline characteristics of patients, surgery information, and 90-day events according to ascites (presence vs. absence).

	All patients (*n* = 482)	No ascites (*n* = 342)	Ascites (*n* = 140)	*p*
**Baseline characteristics**				
**Age** (years)	66 (57–75)	66 (56–76)	66 (58–75)	0.744
**Male sex**, n (%)	318 (66.0)	224 (65.5)	94 (67.1)	0.846
**ASA**, n (%)				<0.001
II	61 (12.7)	61 (17.8)	0 (0)	
III	348 (72.2)	248 (72.5)	100 (71.4)	
IV	73 (15.2)	33 (9.7)	40 (28.6)	
**Etiology of liver disease**, n (%)				
Viral	159 (33.0)	124 (36.3)	35 (25.0)	0.011
Alcohol	244 (50.6)	158 (46.2)	86 (61.4)	0.003
MASLD	54 (11.2)	41 (12.0)	13 (9.3)	0.393
Other	25 (5.2)	19 (5.6)	6 (4.3)	0.749
**Creatinine** (mg/dL) (n = 481)	0.9 (0.7–1.2)	0.9 (0.7–1.1)	0.9 (0.7–1.3)	0.432
**Total bilirubin** (mg/dL) (n = 436)	0.8 (0.5–1.4)	0.7 (0.5–1.2)	1.1 (0.8–2.1)	<0.001
**Albumin** (g/dL) (n = 429)	4.0 (3.2–4.3)	4.2 (3.6–4.4)	3.3 (2.7–3.9)	<0.001
**INR** (n = 481)	1.2 (1.1–1.3)	1.2 (1.1–1.3)	1.3 (1.2–1.4)	<0.001
**Platelet count** (× 10^3^/uL)	129 (92–187)	137 (99–192)	110 (80–170)	<0.001
**MELD-Na** (n = 436)	12 (8–16)	11 (8–14)	14 (10–20)	<0.001
**CTP class**, n (%) (n = 420)				
A	285 (70.2)	245 (88.8)	40 (30.8)	<0.001
B	96 (23.7)	30 (10.9)	66 (50.8)	
C	25 (6.2)	1 (0.4)	24 (18.5)	
**LSM** (kPa) (n = 197)	15.7 (11.2–23.6)	15.7 (11.2–23.6)	−	−
**Splenomegaly + thrombocytopenia** (n = 475)	190 (40.0)	115 (34.0)	75 (54.7)	<0.001
**GOV**, n (%) (n = 403)	245 (60.8)	145 (52.7)	100 (78.1)	<0.001
**Surgery information**				
**Type of surgery**, n (%)				0.002
Abdominal	197 (40.9)	155 (45.3)	42 (30.0)	
Non-abdominal	285 (59.1)	187 (54.7)	98 (70.0)	
**Emergent surgery**, n (%)	191 (39.6)	114 (33.3)	77 (55.0)	<0.001
**Oncologic surgery**, n (%)	62 (12.9)	52 (15.2)	10 (7.1)	0.016
**Period of surgery,** n (%)				0.189
2010–2014	232 (47.0)	151 (44.2)	71 (50.7)	
2015–2019	262 (53.0)	191 (55.9)	69 (49.3)	
**VOCAL-Penn’s 30-day predicted mortality** (%)	1.2 (0.3–4.3)	0.6 (0.2–2.5)	4.5 (1.4–10.1)	<0.001
**Events and mortality at 90 days**				
**AKI,** n (%) (n = 446)	148 (33.2)	84 (27.0)	64 (47.4)	<0.001
AKI-1A	37 (8.3)	23 (7.4)	14 (10.4)	<0.001
AKI-1B	42 (9.4)	28 (9.0)	14 (10.4)	
AKI-2	35 (7.8)	16 (5.1)	19 (14.1)	
AKI-3	34 (6.3)	17 (5.5)	17 (12.6)	
**Ascites**, n (%)				<0.001
Worsening	23 (4.8)	-	23 (16.4)	-
New-onset ascites	65 (14.5)	65 (19.0)	-	-
Improvement	27 (5.6)	-	27 (19.3)	-
**Other first hepatic decompensation**, n (%)	15 (3.1)	6 (1.8)	9 (6.4)	<0.001
**Bacterial peritonitis**, n (%)	53 (11.0)	21 (6.1)	32 (22.9)	<0.001
**Any LREs**, n (%)	160 (33.2)	71 (20.8)	89 (63.6)	<0.001
**Death**, n (%)	46 (9.5)	22 (6.4)	24 (17.1)	<0.001

BMI: Body mass index; ASA: American Society of Anesthesiologists Physical Status Classification System.; MASLD: metabolic dysfunction–associated steatotic liver disease; INR: International Normalized Ratio; MELD: Model for End-Stage Liver Disease; CTP: Child–Turcotte–Pugh; ACLD: advanced chronic liver disease; LSM: liver stiffness measurement; GOV: gastroesophageal varices; LREs: liver-related events; AKI: acute kidney injury.

**Table 2:** Baseline characteristics of patients, surgery information, and 90-day events according to the emergency of surgery (emergent vs. elective).

	Elective (*n* = 291)	Emergent (*n* = 191)	*p*
**Baseline characteristics**			
**Age** (years)	67 (57–74)	65 (55–78)	0.896
**Male sex**, n (%)	190 (65.3)	128 (67.2)	0.696
**ASA**, n (%)			<0.001
II	39 (13.4)	22 (11.5)	
III	224 (77.0)	124 (64.9)	
IV	28 (9.6)	45 (23.6)	
**Etiology of liver disease**, n (%)			
Viral	98 (33.7)	61 (31.9)	0.474
Alcohol	137 (47.1)	107 (56.0)	0.035
MASLD	37 (12.7)	17 (8.9)	0.194
Other	18 (6.5)	6 (3.1)	0.174
**Creatinine** (mg/dL) (n = 481)	0.9 (0.7–1.1)	0.9 (0.7–1.3)	0.460
**Total bilirubin** (mg/dL) (n = 436)	0.7 (0.5–1.1)	1.1 (0.6–2.0)	<0.001
**Albumin** (g/dL) (n = 429)	4.1 (3.5–4.5)	3.5 (2.9–4.1)	<0.001
**INR** (n = 481)	1.1 (1.1–1.2)	1.3 (1.1–1.5)	<0.001
**Platelet count** (×10^3^/uL)	134 (100–189)	114 (84–183)	0.010
**MELD-Na** (n = 436)	10 (8–14)	14 (11–19)	<0.001
**CTP**, n (%) (n = 406)			
A	202 (80.5)	83 (53.6)	<0.001
B	42 (16.7)	54 (34.8)	
C	7 (2.8)	18 (11.6)	
**Ascites 30 days before surgery**, n (%)			<0.001
Grade 2	41 (14.1)	27 (14.1)	
Grade 3	22 (7.6)	50 (26.2)	
**LSM** (kPa) (n = 197)	15.5 (11.2–23.4)	15.9 (11.5–25.5)	0.984
**Splenomegaly + thrombocytopenia** (n = 475)	113 (39.7)	77 (40.5)	0.848
**GOV**, n (%) (n = 403)	143 (58.4)	102 (64.6)	0.214
**Surgery information**			
**Type of surgery**, n (%)			0.091
Abdominal	110 (37.8)	104 (54.5)	
Non-abdominal	181 (62.2)	87 (45.5)	
**Oncologic surgery**, n (%)	56 (19.2)	6 (3.1)	<0.001
**Period of surgery,** n (%)			0.189
2010–2014	127 (43.6)	95 (49.7)	
2015–2019	164 (56.4)	96 (50.3)	
**VOCAL-Penn’s 30-day predicted mortality** (%)	0.5 (0.2–2.0)	3.6 (1.4–11.0)	<0.001
**Events and death at 90 days**			
**AKI,** n (%) (N = 446)	63 (24.2)	85 (45.7)	<0.001
AKI-1A	15 (5.8)	22 (11.8)	<0.001
AKI-1B	19 (7.3)	23 (12.4)	
AKI-2	18 (6.9)	17 (9.1)	
AKI-3	11 (4.2)	23 (12.4)	
**Ascites**, n (%)			
Worsening	9 (3.1)	14 (7.3)	0.033
New-onset ascites	25 (8.6)	40 (20.9)	<0.001
Improvement	18 (6.2)	9 (4.7)	0.491
**Other first hepatic decompensation**, n (%)	2 (0.7)	13 (6.8)	<0.001
**Bacterial peritonitis**, n (%)	19 (6.5)	34 (17.8)	<0.001
**Any LREs**, n (%)	53 (18.2)	107 (56.0)	<0.001
**Death**, n (%)	16 (5.5)	30 (15.7)	<0.001

ASA: American Society of Anesthesiologists Physical Status Classification System.; MASLD: metabolic dysfunction–associated steatotic liver disease; INR: International Normalized Ratio; MELD: Model for End-Stage Liver Disease; CTP: Child–Turcotte–Pugh; LSM: liver stiffness measurement; GOV: gastroesophageal varices; LREs: liver-related events; AKI: acute kidney injury.

## Data Availability

Data are unavailable due to ethical restrictions.

## Supplementary Materials

The following supporting information can be downloaded at https://www.mdpi.com/article/10.3390/jcm14041077/s1, Table S1: Baseline characteristics according to the period (2010-2014 vs. 2015-2019); Table S2: Variables related to 30-day mortality according to type of surgery (abdominal vs. non-abdominal).
