# Supplementary material for: Clinical Ascites and Emergency Procedure as Determinants of Surgical Risk in Patients with Advanced Chronic Liver Disease
Source: J Clin Med. 2025 Feb 8;14(4):1077. doi: 10.3390/jcm14041077 (PMC11856016; doi:10.3390/jcm14041077)
Supplement: Supplementary file 1 [file jcm-14-01077-s001.zip › JCM_Supplementary Table S1.pdf]

	2010 - 2014 (N=222)	2015 - 2019 (N=260)	p
**BASELINE CHARACTERISTICS**			
**Age** (years)	64 (53 – 75)	68 (59 – 76)	0.004
**Male sex**, n (%)	140 (63.1)	178 (68.5)	0.212
**ASA**, n (%)			0.830
II	28 (12.6)	33 (12.7)	
III	158 (71.2)	190 (73.1)	
IV	36 (16.2)	37 (14.2)	
**Etiology of liver disease**, n (%)			
Viral	77 (34.7)	82 (31.5)	0.410
Alcohol	113 (50.9)	131 (50.4)	0.758
MASLD	19 (8.6)	35 (13.5)	0.089
Other	13 (5.9)	12 (4.6)	0.547
**Creatinine** (mg/dl) (n=481)	0.9 (0.7 – 1.1)	0.9 (0.7 – 1.2)	0.308
**Total bilirubin** (mg/dl) (n=436)	0.9 (0.6 – 1.5)	0.7 (0.5 – 1.3)	0.004
**Albumin** (g/dl) (n=429)	3.9 (3.2 – 4.3)	4 (3.3 – 4.4)	0.034
**INR** (n=481)	1.2 (1.1 – 1.4)	1.1 (1.1 – 1.3)	<0.001
**Platelet count** ($\cdot 10^3$/uL)	119 (87 – 176)	140 (95 – 198)	0.011
**MELD-Na** (n=436)	12 (9 – 17)	11 (8 – 15)	0.021
**Child-Pugh Class**, n (%) (n=406)			0.186
A	121 (65.8)	164 (73.9)	
B	49 (26.6)	47 (21.2)	
C	14 (7.6)	11 (4.9)	
**Ascites 30 days before surgery**, n (%)			0.417
Controlled with diuretics	34 (15.3)	34 (13.1)	
Clinical ascites	37 (16.7)	35 (13.5)	
<u>**LSM** (kPa) (n=197)</u>	<u>17.5 (11.2 – 26.3)</u>	<u>15.3 (11.3 – 22.6)</u>	<u>0.252</u>
**Splenomegaly + thrombocytopenia** (n=475)	96 (44.4)	94 (36.3)	0.071
<u>**GOV**</u>, n (%) (n=403)	111 (58.4)	134 (62.9)	0.357
**SURGERY INFORMATION**			
**Type of surgery**, n (%)			0.002
Abdominal	107 (48.2)	90 (34.6)	
Non-abdominal	115 (51.8)	170 (65.4)	
**Emergent surgery**, n (%)	95 (42.8)	96 (36.9)	0.189
<u>**Oncologic surgery**, n (%)</u>	<u>32 (14.4)</u>	<u>30 (11.5)</u>	<u>0.337</u>
<u>**VOCAL-Penn's 30-day predicted mortality** (%)</u>	<u>1.4 (0.4 – 3.8)</u>	<u>1.0 (0.2 – 5.1)</u>	<u>0.240</u>
**LREs AND MORTALITY AT 90 DAYS**			
**AKI,** n (%) (N=446)	70 (33.5)	78 (32.9)	0.896
AKI-IA	18 (8.6)	19 (8.0)	0.974
AKI-IB	18 (8.6	24 (10.1)	
AKI-II	17 (8.1)	18 (7.6)	
AKI-III	17 (8.1)	17 (7.2)	
**Ascites**, n (%)			
Worsening	13 (5.9)	10 (3.9)	0.302
New-onset ascites	38 (17.1)	27 (10.4)	0.031
<u>Improvement</u>	<u>16 (7.2)</u>	<u>11 (4.2)</u>	<u>0.157</u>

**Bacterial peritonitis**, n (%)	35 (15.8)	18 (6.9)	0.002
**Any LREs**, n (%)	90 (40.5)	70 (26.9)	0.002
**Death**, n (%)	30 (13.5)	16 (6.2)	0.006

Supplementary Table S1: Baseline characteristics of patients, surgeries, and 90-day clinical events according to the period (2010-2014 vs. 2015-2019).

ASA: American Society of Anesthesiologists Physical Status Classification System; MASLD: Metabolic dysfunction–associated steatotic liver disease; INR: International Normalized Ratio; MELD: Model for End-Stage Liver Disease; CTP: Child-Tucotte-Pugh; LSM: liver stiffness measurement; GOV: gastroesophageal varices; LREs: liver-related events; AKI: acute kidney injury.
