# Supplementary material for: Clinical Ascites and Emergency Procedure as Determinants of Surgical Risk in Patients with Advanced Chronic Liver Disease
Source: J Clin Med. 2025 Feb 8;14(4):1077. doi: 10.3390/jcm14041077 (PMC11856016; doi:10.3390/jcm14041077)
Supplement: Supplementary file 1 [file jcm-14-01077-s001.zip › JCM_Supplementary Table S2.pdf]

	ABDOMINAL SURGERY			NON-ABDOMINAL SURGERY		
	**Live patients (n=181)**	**Deceased patients (n=16)**	**p**	**Live patients (n=270)**	**Deceased patients (n=15)**	**p**
**Age** (years)	65 (55 – 74)	66 (61 – 78)	0.288	66 (57 – 75)	78 (70 – 83)	0.002
**Male sex**, n (%)	121 (66.9)	13 (81.3)	0.237	178 (65.9)	6 (40.0)	0.041
**ASA IV**, n (%)	33 (18.2)	12 (75.0)	<0.001	64 (23.7)	9 (60.0)	0.002
**Etiology of liver disease**, n (%)						
Viral	53 (29.3)	3 (17.8)	0.371	95 (35.2)	8 (53.3)	0.367
Alcohol	97 (53.6)	8 (50.0)	0.838	135 (50.0)	4 (26.7)	0.180
MASLD	23 (12.7)	4 (25.0)	0.171	25 (9.2)	2 (13.3)	0.600
Other	8 (4.4)	1 (6.2)	0.391	15 (5.6)	1 (6.7)	0.803
**Creatinine** (mg/dl) (n=481)	0.9 (0.7 – 1.1)	1.3 (0.7 – 1.5)	0.128	0.9 (0.7 – 1.1)	1.2 (1.0 – 1.8)	0.001
**Total bilirubin** (mg/dl) (n=436)	0.8 (0.5 – 1.4)	1.9 (1.2 – 3.7)	<0.001	0.8 (0.5 – 1.3)	1.8 (0.9 – 2.6)	0.003
**Albumin** (g/dl) (n=429)	4.0 (3.3 – 4.4)	2.9 (2.3 – 3.6)	<0.001	4.0 (3.3 – 4.4)	3.2 (2.6 – 4.2)	0.037
**INR** (n=481)	1.2 (1.1 – 1.3)	1.5 (1.2 – 1.9)	0.002	1.2 (1.1 – 1.3)	1.3 (1.2 – 1.4)	0.005
**Platelet count** ($\cdot 10^3$/uL)	134 (93 – 186)	100 (72 – 131)	0.060	129 (94 – 193)	102 (67 – 159)	0.071
**Ascites**, n (%)	34 (18.8)	8 (50.0)	0.003	91 (33.7)	7 (46.7)	0.304
**GOV**, n (%) (n=403)	79 (51.0)	10 (90.9)	0.010	147 (65.3)	9 (75.0)	0.492
**Hypertension**, n (%)	77 (42.5)	9 (56.3)	0.289	130 (48.2)	6 (40.0)	0.539
**Diabetes**, n (%)	56 (30.9)	7 (43.8)	0.292	77 (28.5)	4 (26.7)	0.877
**Obesity (BMI ≥ 30 kg/m²)** , n (%) (n=454)	53 (32.3)	4 (25.0)	0.548	75 (31.5)	3 (33.3)	0.908
**Chronic kidney disease**, n (%)	23 (12.7)	3 (18.8)	0.494	45 (16.7)	6 (40.0)	0.022
**Peripheral vascular disease**, n (%)	6 (3.3)	3 (18.8)	0.005	28 (10.4)	1 (6.7)	0.644
**Acute myocardial infarction**, n (%)	5 (2.8)	3 (18.8)	0.002	17 (6.3)	0 (0)	0.316
**Congestive heart failure**, n (%)	7 (3.9)	3 (18.8)	0.009	15 (5.6)	1 (6.7)	0.856
**Active cancer**, n (%)	48 (26.5)	6 (37.5)	0.345	42 (15.6)	2 (13.3)	0.817
**Open abdominal surgery**, n (%)	98 (54.1)	15 (93.8)	0.002	–	–	–
**Emergent surgery**, n (%)	76 (42.0)	11 (68.8)	0.039	95 (35.2)	9 (60.0)	0.052
**Period of surgery**, n (%)			0.718			0.033
2010 – 2014	99 (54.7)	8 (50.0)		105 (38.9)	10 (66.7)	
2015 – 2019	82 (45.3)	8 (50.0)		165 (61.1)	5 (33.3)	

Supplementary Table S2. Univariate analysis of variables related to 30-day mortality according to type of surgery (abdominal vs. non-abdominal). ASA: American Society of Anesthesiologists Physical Status Classification System; MASLD: Metabolic dysfunction–associated steatotic liver disease; INR: International Normalized Ratio; GOV: gastroesophageal varices; BMI: body mass index.
